# Facile Synthesis of Polyaniline Nanotubes with Square Capillary Using Urea as Template

**DOI:** 10.3390/polym9100510

**Published:** 2017-10-14

**Authors:** Shuhua Pang, Weiliang Chen, Zhewei Yang, Zheng Liu, Xin Fan, Dong Fang

**Affiliations:** 1 Key Laboratory of New Processing Technology for Nonferrous Metal and Materials of Ministry of Education, College of Materials Science & Engineering, Guilin University of Technology, Guilin 541004, China; pang_s_h@163.com (S.P.); chenweilliang@163.com (W.C.); zheweiyangglut @163.com (Z.Y.); zhengliu@163.com (Z.L.); 2 BengBu Center of Product Quality Supervising and Inspection, BengBu 233000, China; 3 Key Lab of Green Processing and Functional Textiles of New Textile Materials, Ministry of Education, College of Material Science and Engineering, Wuhan Textile University, Wuhan 430700, China

**Keywords:** polyaniline, nanotube, urea, template, morphology, electrochemical performance

## Abstract

Polyaniline nanotubes were successfully synthesized by a facile in situ chemical oxidative polymerization method using urea as soft template. When the urea/aniline molar ratio is 3:1, the as-prepared nanotubular polyaniline (PANI-3) shows regular and uniform square capillaries, which provides a high electrode/electrolyte contact, easy ion diffusion and enhanced electroactive regions during the electrochemical process, leading to weak internal resistance and improved electrochemical performance. The PANI-3 sample exhibits a high specific capacitance of 405 F/g at current density of 0.2 A/g, and PANI only has a specific capacitance of 263 F/g. At current density of 1 A/g, the capacitance of PANI-3 is still 263 F/g (64.9% of the capacitance at 0.2 A/g). Such a PANI-3 nanotube, with regular and uniform capillary, is a promising electrode material for high-performance supercapacitors.

## 1. Introduction

Supercapacitor, also known as electrochemical capacitor (EC), is one of the most promising energy storage devices, and has attracted enormous attention [1,2] in recent years because of its unique properties, including high specific power, excellent cycle stability and environmental friendliness [3,4], compared to the batteries and conventional electrolytic capacitors [5,6]. Electrode materials, namely, active materials in electrodes of supercapacitors, have significant effects on the capability, delivery rates, and efficiency of energy storage devices [7]. In the past decades, conducting polymers (CPs), especially nanostructured CPs, which provide higher specific surface area, and relatively shorter ion diffusion and migration paths, and mitigated mechanical deformation than bulk ones, have exhibited attractive performance in energy storage applications [8,9,10]. Nanostructured polyaniline (PANI), one of the nanostructured CPs, has been extensively investigated as an active electrode material for high-performance supercapacitors, due to its desirable properties, such as high charge storage ability, high conductivity, excellent environmental stability, good redox reversibility, facile synthesis, and low cost [10,11], etc. The electrochemical properties of the electrode materials are believed to depend strongly on the morphology of active materials [12]. To achieve superior performance, PANI materials with various morphologies were prepared, including nanotubes [13], nanowires [14], nanofibers [15], and nanospheres [16]. Employing a chemical bath deposition method, Dhawale et al. [17] synthesized PANI nanograins with diameter of 20–30 nm, which delivers a maximum specific capacitance of 503 F/g. Ran et al. [18] fabricated polyaniline nanotube supercapacitors using salicylic acid as the template and doping agent, but the shape of the reported nanotube is not regular and obvious, which seriously affects the specific capacitance of PANI.

Recently, both hard and soft template methods have been widely employed as a structure-directing agent to prepare PANI nanotubes with special size and anticipative morphology for high-performance supercapacitor electrode materials [11,19,20,21,22]. However, the approaches of hard templates require post treatments to remove the template, which make it rather difficult to retain the ordered structure after template removal [11,22,23,24]. Therefore, the soft template method has been widely adopted to prepare nanostructured PANI through a self-assembly process, due to easy processing and environmental friendliness [19,25,26].

Herein, we report a novel and facile strategy to synthesize nano-structured PANI with nano-tubular particulate morphology. In this process, urea, as soft template, can be aggregated with aniline through H-bonding to form core–shell structured rods, with urea assembly as core and aniline as shell. After in situ polymerization of aniline and the removal of urea, PANI nanotubes with a square capillary channel were obtained. The nanotube structures with high specific surface area provide more active sites for electrode reaction, thus, the prepared PANI nanotubes exhibit high specific capacitance and excellent rate capability. Considering the simplicity of employed method and the availability of urea, as well as the superiority of electrochemical properties, the prepared nanotubular PANI is a promising electrode material for supercapacitor in larger-scale applications.

## 2. Experimental

### 2.1. Synthesis of Polyaniline Nanotubes

Aniline was purchased from Aladdin and was distilled before use. Ammonium persulfate (APS), hydrochloric acid (HCl), and urea (Xilong Chemical Co. Ltd., Shantou, China) were analytical grade, and used as received. A typical experiment process for synthesis of polyaniline nanotubes with square capillary (also known as square porous structure) is shown as follows, according to the reference [26], using urea as the template instead of vitamin C.

Urea (0.9857 g), as the template, was dispersed into 30 mL deionized water, and stirred for 0.5 h at room temperature. Then, 0.5 mL aniline (the molar ratio of urea to aniline is 3) and 2 mL of 1 M HCl were added into the urea aqueous solution, and stirred for 1 h in an ice-water bath to obtain the uniform solution. Next, 10 mL aqueous solution of 1.2498 g APS (molar ratio of aniline to APS = 1:1) was added dropwise into the above solution. The reaction was carried out under vigorous magnetic stirring in an ice-water bath for 10 h. Then, 5 mL of 1 M HCl was added into the resulting suspension to ensure the complete doping of PANI. The product was filtered out, and the resulting precipitate was washed several times with deionized water until the filtrate was neutral. Finally, the product was dried overnight in a vacuum oven at 60 °C, and denoted as PANI-3.

In order to investigate the influence of urea/aniline molar ratio on the capillary morphology, the capillary size and electrochemical properties of PANI nanotubes, the PANI without urea and with different molar ratios of urea to aniline (0.25, 1, 2, 3 and 4) were studied, and the resultant products were denoted as PANI, PANI-0.25, PANI-1, PANI-2, PANI-3, PANI-4, respectively.

### 2.2. Material Characterization

The morphologies of PANI products were observed by scanning electron microscopy (SEM, S4800, Tokyo, Japan), and transmission electron microscopy (TEM, JEM-2100F, Tokyo, Japan). FTIR spectra of the products were recorded on a Nicolet Nexus 670 Fourier transform infrared spectrometer (Nicolet, Madison, WI, USA) in the range of 4000–400 cm^−1^.

### 2.3. Electrochemical Measurements

The electrochemical measurements were performed on CHI660 electrochemical workstation in an electrolyte of 1 M H_2_SO_4_ solution using a three-electrode mode. Working electrode was prepared as follows: mixing PANI, conductive black and polytetrafluorethylene (mass ratio = 8:1:1) to yield a homogeneous slurry under ultrasonic dispersion. The slurry was coated and pressed onto stainless steel mesh (the area is 1 cm^2^, and the mass of active material was about 5~10 mg), then dried in a vacuum oven at 60 °C for 24 h. The reference electrode and counter electrode were saturated calomel electrode (SCE) and platinum electrode, respectively. Typical cyclic voltammetry (CV) curves of the PANI nanotube electrodes were measured between −0.2 and 0.8 V. The galvanostatic charge/discharge (GCD) property was evaluated at the current densities of 0.2, 0.5, 0.7 and 1 A·g^−1^, with cutoff voltage of −0.2–0.8 V. Electrochemical impedance spectroscopy (EIS) tests were conducted at open circuit potential of 0.4 V in the frequency range from 100 kHz to 0.01 Hz with a sinusoidal signal of 5 mV.

All these tests were conducted at room temperature and ambient pressure. The specific capacitance of the electrode was derived from GCD according to the following equation:

*C*_s_ = (*I*·Δ*t*)/(Δ*V*·*m*) (1)

where *C*_s_ is the specific capacitance (F/g), *I* is the current of discharge (A), Δ*t* is the discharge time (s), *m* is the total mass of active material in a single electrode (g), and Δ*V* is the potential range during discharge process (V).

The energy density *E* (Wh·kg^−1^) and power density *P* (W·kg^−1^) can be calculated as

*E* = 0.5·*C_s_*·Δ*V*^2^/3.6 (2)

*P* = 3600*E*/Δ*t* (3)

## 3. Results and Discussion

### 3.1. Structural and Morphology Analysis

**Figure 1:**
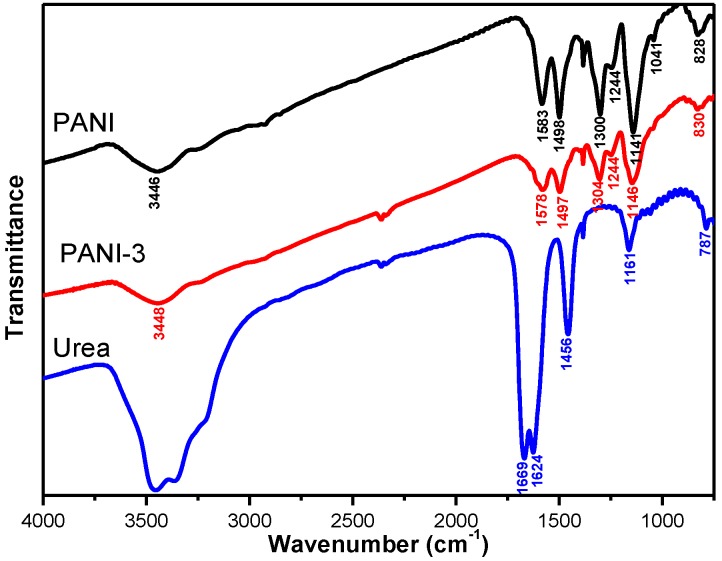
FT-IR spectra of polyaniline (PANI) and PANI-3 nanotubes and urea samples.

The FT-IR spectra are shown in Figure 1. For PANI and PANI-3 nanotubes samples, the typical characteristic peaks, belonging to PANI, can be easily observed [27,28,29]. To be specific, peaks at 1583 cm^−1^ (PANI) and 1578 cm^−1^ (PANI-3) corresponded to the C=C stretching deformation of quinonoid rings, while that at 1498 cm^−1^ (PANI) and 1497 cm^−1^ (PANI-3) are due to the C=C stretching deformation of benzene rings. The peaks at 1300~1304 and 1244 cm^−1^ can be attributed to C–N and C=N stretching of an aromatic amine. The peak at 1141~1146 cm^−1^ can be assigned to the aromatic C–H in-plane bending, and the peak at 828 cm^−1^ is related to the out of plane deformation of C–H in the 1,4-disubstituted benzene ring. PANI and PANI-3 samples show similar feature in FT-IR spectra, implying that urea as soft template does not change the molecular structure of PANI. On the other hand, the characteristic peaks of urea cannot be observed in PANI-3 spectrum, indicating that urea had been removed completely.

**Figure 2:**
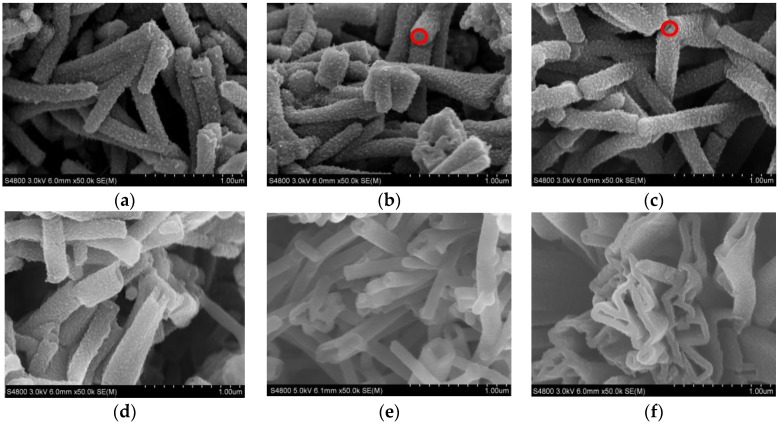
SEM images of PANI nanostructures at different urea/aniline ratios: (**a**) 0:1 (**b**) 1:4 (**c**) 1:1 (**d**) 2:1 (**e**) 3:1 (**f**) 4:1.

**Figure 3:**
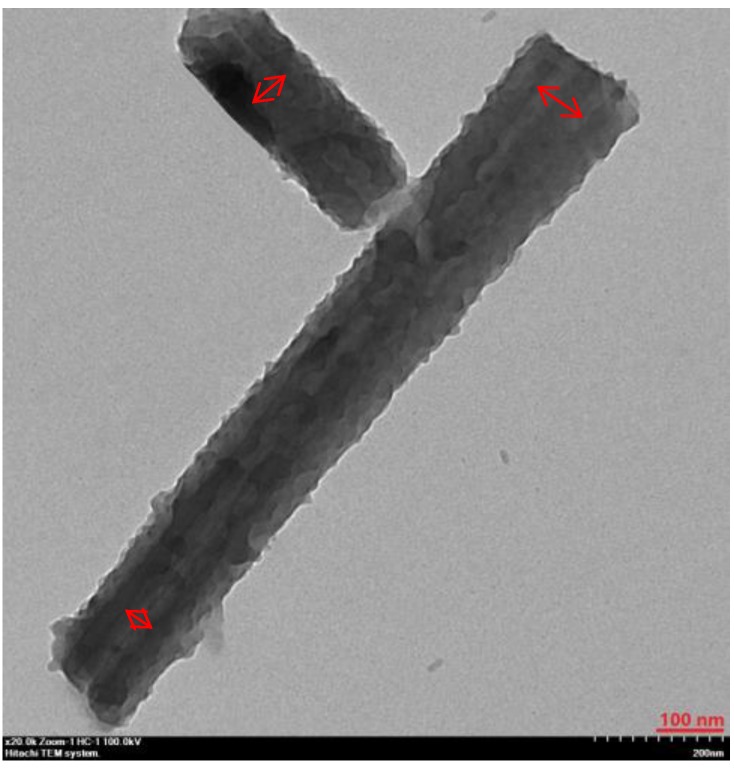
TEM image of PANI-3 nanotube.

Figure 2 shows the SEM images of PANI nanostructures at different urea/aniline ratios. PANI sample, prepared without urea, exhibits a well-defined dense rod-like structures with a diameter of 100–150 nm (Figure 2a). However, PANI-3 sample exhibits regular and uniform nanotube with inner square aperture (Figure 2e), and the average inner and outer diameters of the nanotubes are in the ranges of 60 and 120 nm, respectively. To get a better understanding of the structure, the PANI-3 nanotube was also investigated by TEM. As shown in Figure 3, the side of the square capillary is approximately 70 nm, and the outer diameter is about 140 nm, which is consistent with SEM observations. The large capillary channel can provide space for electrolyte infiltration, leading to more active sites for electrode reaction. Meanwhile, the thinner wall of nanotubes shortens the charge diffusion distance, and thus accelerates the electrode reactions. Both factors are favorable for the realization of fast electrode kinetics.

To explore the forming process and mechanism of PANI nanotubes, the PANI materials were synthesized at different urea/aniline molar ratios, keeping the same aniline/APS molar ratio and reaction temperature. As illustrated in Figure 2, it is found that the other rod-like PANI products exhibit irregular rod-like shape with small hollow structures when the urea/aniline molar ratio is 1:4 (Figure 2b). With the increase of urea/aniline molar ratio, the nanostructured PANI products become more homogenous and regular in morphology, as well as increasing in capillary size (Figure 2c,d). Interestingly, it can be observed that the capillary channel of the nanotubes appear to be a rectangular shape when the ratio increases to 3:1 (Figure 2e). However, when the ratio increases to 4:1, considerable tabular nanotube aggregates are observed instead (Figure 2f). These results suggest that the urea/aniline ratio has a significant effect on the morphology of the PANI nanostructures.

**Scheme 1:**
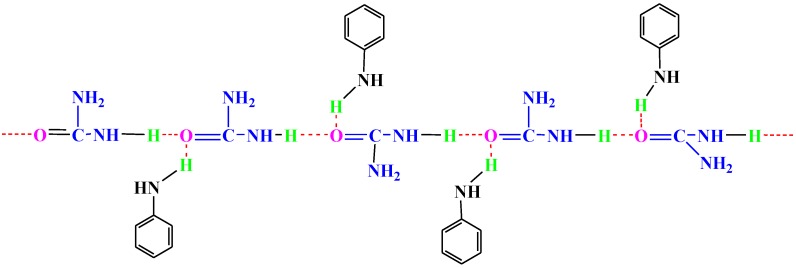
Schematic diagram of molecule self-assembly of aniline and urea throng hydrogen bond.

**Scheme 2:**
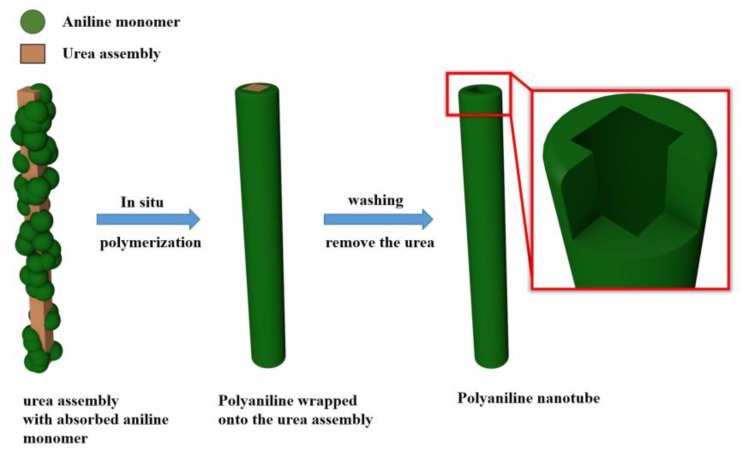
Schematic illustration of the synthesis of polyaniline nanotube.

It is well known that hydrogen bond plays a crucial role in the self-assembly process. With regard to the formation mechanism of the PANI nanotubes using urea as the template, it could be proposed that the intermolecular hydrogen bond might be a driving force for self-assembly of PANI nanostructures: first, the urea is self-assembled by means of the intermolecular hydrogen bond derived from –C=O and –NH_2_ group in the urea chain; then, aniline monomers gather around urea self-assembly; then, the intermolecular hydrogen bond through –C=O group of urea and amine of aniline monomer is formed; finally, aniline monomers are polymerized into PANI, as shown in Scheme 1 and Scheme 2. When the urea/aniline ratio is less than 1:1, a small number of urea micelles are formed, and a plenitude of aniline monomers gather around urea micelles, and therefore, PANI nanostructures show small and narrow capillary channels, as illustrated in Figure 2a–c. As the ratio of urea/aniline gradually increases, more and more urea micelles are formed, and part of the urea micelles gather together, then aniline monomers adsorbed around urea micelles are polymerized into PANI nanostructures with larger pores. It can be clearly found from SEM data that the mechanism of PANI nanotube is assembled from nanoparticles. Especially when the ratio of urea/aniline ratio increases up to 3:1, it is suitable to form PANI nanostructure with uniform and square capillary. With the further increasing of urea/aniline ratio to 4:1 or more, excessive urea polymer chains gather together so that the resulting PANI nanostructures exhibit irregular and tabular capillary, which reduces the electrochemical performance.

### 3.2. Electrochemical Properties

**Figure 4:**
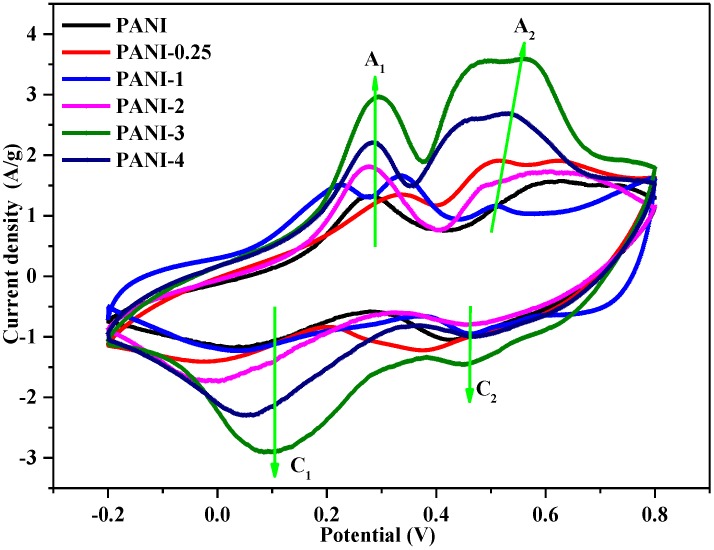
CV curves of nanostructured PANI samples at scan rate of 5 mV/s.

The CV measurement results of nanostructured PANI electrodes are shown in Figure 4. It is observed that all nanostructured PANI samples show similar CV curves, and two couples of redox peaks could be observed, resulting in the pseudocapacitance. Specifically, peaks C_1_/A_1_ were attributed to the redox transition between leucoemeraldine (semiconducting state) and polaronic emeraldine (conducting state), and peaks C_2_/A_2_ were associated with the transformation between emeraldine and pernigraniline, exhibiting the electrochemical behavior of a Faradic capacitor [30,31,32]. The CV curve area of PANI-3 nanotubes is larger than that of the other nanostructured PANI materials, which demonstrates that the electrochemical performance of PANI-3 nanotubes is significantly improved owing to the larger surface area of nanotube relative to nanorod. On the other hand, PANI-3 nanotubes possess higher redox current density than that of PANI nanorods, suggesting fast electrode reaction kinetics.

**Figure 5:**
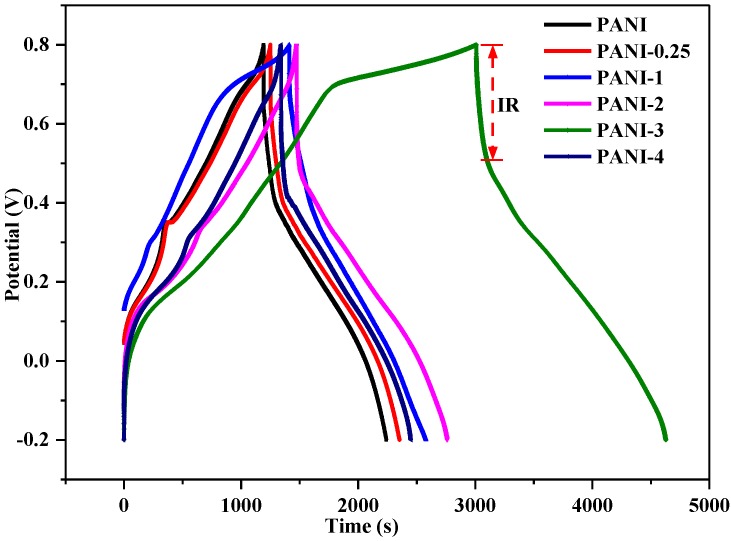
Galvanostatic charge/discharge (GCD) curves of nanostructured PANI samples at current density of 0.2 A/g.

**Figure 6:**
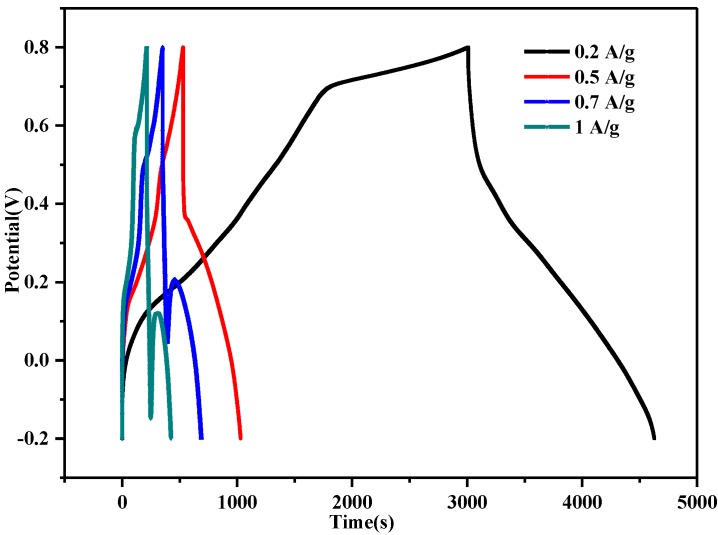
GCD curves of PANI-3 at various current densities.

To further assess the electrochemical performance of PANI nanostructures, GCD measurement was conducted. Figure 5 and Figure 6 show the GCD curves of nanostructured PANI samples at current densities of 0.2 A/g, and PANI-3 at various current densities from 0.2 to 1 A/g, respectively. All curves exhibit triangle shape, but partially deviate from linearity, owing to the contribution of pseudocapacitance [30], revealing that the potential of charge/discharge is a linear response to time, and exhibits good capacitance behavior and excellent reversibility [17,33]. From Figure 5, it could be apparently observed that PANI-3 nanotubes distinctly show larger discharge times as compared with the other PANI products, indicating that PANI-3 possesses higher specific capacitance. This is mainly due to the fact that PANI-3 nanotubes with regular and uniform square capillaries possess highest specific surface area than the other nanostructured PANI products [34]. The specific capacitance (*C*_s_) was calculated according to Equation (1), and illustrated in Table 1. The specific capacitance of various PANI nanostructures samples are 263, 276, 292, 322, 405 and 278 F/g for PANI, PANI-0.25, PANI-1, PANI-2, PANI-3, and PANI-4, respectively. On the other hand, the IR drop can be used to measure the equivalent series resistance (inner resistance) of the electrode, which has an important influence on the supercapacitor performance. In general, lower IR drop means better conductivity and supercapacitive properties. As shown in Figure 5, there is an IR drop at the beginning of the discharge curve of all the PANI. PANI-3 nanotubes have a lower IR drop than the other nanostructured PANI materials, thus suggesting that PANI-3 nanotubes have a better supercapacitive performance. It may be mainly ascribed to the larger specific surface area of PANI-3 nanotubes, resulting from their well-defined and regular rectangular pore tubular nanostructures. It is generally known that nano-sized PANI can reduce the diffusion path, enhance the electroactive regions, and further improve the electrochemical performance, which was necessary for the electron/charge transport in electrodes, and at the electrode/electrolyte interface [2].

The charge–discharge curves of the PANI nanotube electrode materials at various current densities from 0.2 to 1 A/g are used to evaluate the rate performance, and the specific capacitance calculated from Figure 6 was illustrated in Table 2. When the current densities were 0.5 and 0.7 A/g, the specific capacitance decreased to 313 and 295 F/g, respectively, which is due to the fact that the speed of ion diffusion and redox reaction cannot catch up with the larger current density [35]. Even at 1 A/g, the capacitance was still 263 F/g (64.9% of the capacitance at 0.2 A/g), which demonstrates the good rate performance of PANI nanotubes. The rate performance is of great importance for supercapacitor electrode materials to provide high power density in practical applications.

**Figure 7:**
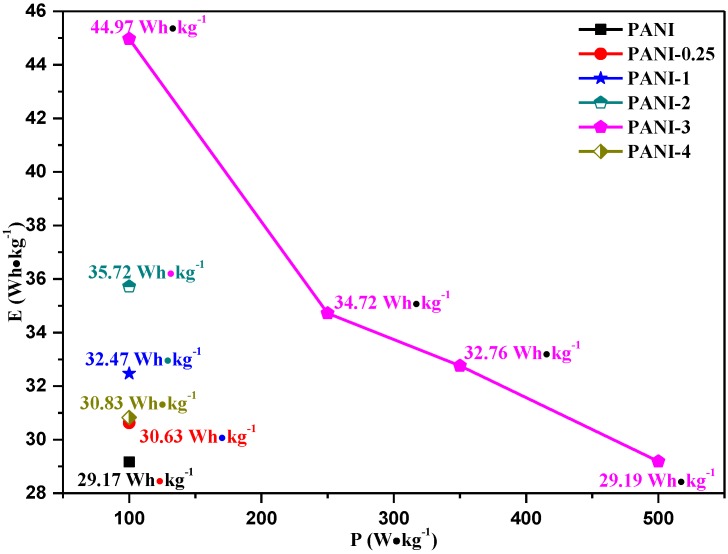
Energy density and power density of nanostructured PANI samples.

In order to demonstrate the overall performance of various nanostructured PANI samples, the Ragone plots were shown in Figure 7. The maximum energy density for PANI-3 nanotubes sample is 44.97 Wh·kg^-1^ (at a power density of 100 W·kg^-1^) and the highest power density is 500 W·kg^-1^ (at an energy density of 29.19 Wh·kg^-1^), respectively. At a power density of 100 W·kg^-1^, the energy density is 29.17, 30.63, 32.47, 35.72, 44.97, and 30.83 Wh·kg^-1^ for PANI, PANI-0.25, PANI-1, PANI-2, PANI-3, and PANI-4, respectively. The Ragone plots demonstrated that PANI-3 nanotubes with square capillary had better electrochemical performance than the other nanostructured PANI materials.

**Figure 8:**
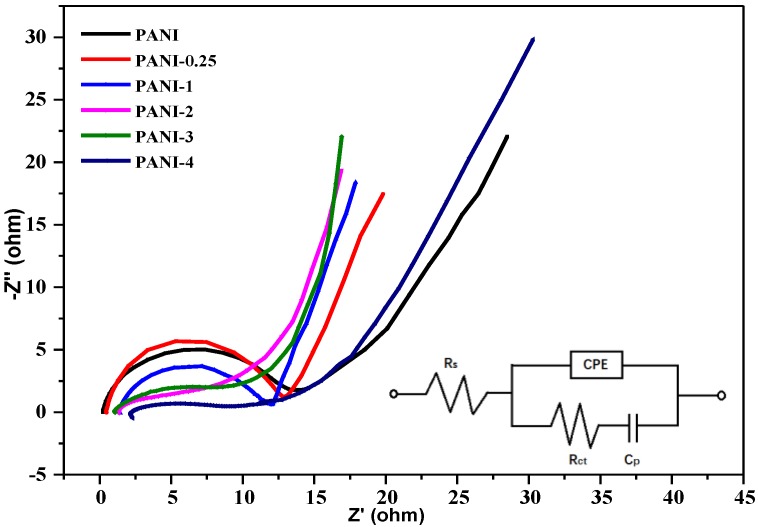
Nyquist plots of nanostructured PANI samples.

EIS was carried out to further study the kinetics of the electrode process, that is, the interaction between electrode and electrolyte. EIS of various nanostructured PANI samples was analyzed by means of Nyquist plots, and the results are displayed in Figure 8. All the plots exhibit two segments, which consist of an approximate semicircle at high-frequency region and a slope line (Warburg region) at low-frequency region, indicating that the electrode process is in the control of both charge transfer and diffusion processes [36,37]. The interfacial charge transfer resistance (*R*_ct_) between electrode and electrolyte is responsible for the generation of the incomplete semicircle [38,39]. When the urea/aniline ratio is less than 1, an apparent semicircular shape at the high frequency region could be observed. However, when the urea/aniline ratio is 2 or even higher, an incomplete semicircle appeared at the high frequency, indicating that PANI nanostructures with larger pore size have lower interfacial charge-transfer resistance through the creation of more excellent electrode/electrolyte contact resulting from the nanotubular structures of PANI products [26]. PANI nanotubes show lower *R*_ct_ than the other PANI nanostructures, which implies that the tubular nanostructure could efficiently reduce the inner resistance of PANI products. The slopes at the low frequency region corresponding to the pseudocapacitor of nanostructured PANI become steeper and steeper with the increase of urea/aniline ratio up to 3:1, thereby suggesting shorter ion-diffusion pathways, facilitating charge transfer, and easier ion-diffusion process of PANI nanotubes [40], indicating better pseudocapacitive behavior in the PANI-3 electrode material with regular pore structure. Nevertheless, the capillaries of PANI-4 products become distorted and irregular, leading to higher interfacial charge-transfer resistance and lower pseudocapacitive behavior relative to PANI-3 nanotubes.

## 4. Conclusions

In summary, PANI nanotubes with uniform square capillaries were successfully synthesized via a facile in situ chemical oxidative polymerization method using urea as soft template. The urea/aniline molar ratio has a significant effect on the morphologies of nanostructured PANI materials, consequently affecting the electrochemical properties strongly. The nanotube with regular rectangle capillaries in PANI-3 products facilitates electron/charge transfer of electrode material, and prompts ion diffusion of the electrolyte during the charge–discharge process, further improving the specific capacitance derived from the advancement of the specific surface area of PANI materials. Consequently, PANI-3 nanotube shows an excellent electrochemical performance and a high specific capacitance, which gives it significant potential application as a promising electrode material for high-performance supercapacitors.

## Figures and Tables

**Table 1:** Specific capacitance of nanostructured PANI samples at current density of 0.2A/g.

Sample	PANI	PANI-0.25	PANI-1	PANI-2	PANI-3	PANI-4
Specific capacitance (F/g)	263	276	292	322	405	278

**Table 2:** Specific capacitance of PANI-3 at different current densities.

Current Density (A/g)	0.2	0.5	0.7	1.0
Specific capacitance (F/g)	405	313	295	263
